# Sarcopenia as an Independent Predictor of Poorer Functional Outcomes in Patients Undergoing Post-hip Fracture Rehabilitation

**DOI:** 10.7759/cureus.91105

**Published:** 2025-08-27

**Authors:** Hideaki Sawamura, Hirokazu Inoue, Yukinori Hayashi, Masaaki Masubuchi, Katsushi Takeshita

**Affiliations:** 1 Department of Orthopaedics, Jichi Medical University, Shimotsuke, JPN; 2 Rehabilitation Center, Jichi Medical University Hospital, Shimotsuke, JPN; 3 Department of Orthopaedic Surgery, Shiobara Spring Hospital, Nasushiobara, JPN

**Keywords:** balance, hip fracture, pain, rehabilitation, sarcopenia, walking speed

## Abstract

Background: Sarcopenia is a critical geriatric syndrome associated with an increasing global aging population and a reduced quality of life. While sarcopenia is known to negatively impact functional ability, its specific effects on rehabilitation outcomes following hip fracture surgery remain to be fully elucidated. The purpose of this study was to investigate the influence of sarcopenia on physical function and recovery trajectory during rehabilitation after hip fracture surgery.

Methods: This retrospective, single-center observational study was conducted at Shiobara Spring Hospital in Nasushiobara, Tochigi, Japan, and included 128 patients who underwent rehabilitation after hip fracture surgery. Participants were classified into two groups based on the presence of sarcopenia, diagnosed according to the Asian Working Group for Sarcopenia 2019 (AWGS 2019) criteria. The primary outcomes were physical function and pain, evaluated by the Berg Balance Scale (BBS), Numerical Rating Scale (NRS) for pain, walking speed, and Barthel Index, measured on both admission and at discharge. We performed a multivariate logistic regression analysis to assess the independent association between sarcopenia and discharge outcomes.

Results: Sarcopenia was prevalent in 66.4% of the patient population. At admission, the sarcopenia group had significantly worse scores on all measures compared to the non-sarcopenia group (BBS, NRS, walking speed, Barthel Index, and calf circumference). Following rehabilitation, both groups showed significant improvements in their functional scores. The multivariate analysis revealed that sarcopenia was a significant independent predictor of lower functional outcomes at discharge. Notably, sarcopenia was strongly associated with a substantially slower walking speed at discharge (odds ratio (OR) 28.635, 95% confidence interval (CI) 4.780-171.552, P < 0.001) and a smaller calf circumference (OR 1.429, 95% CI 1.145-1.784, P = 0.002).

Conclusion: Sarcopenia is a significant independent predictor of poorer physical outcomes at discharge for patients with hip fractures, with a particularly strong association with reduced walking speed. These findings underscore the clinical importance of early sarcopenia diagnosis and the need for targeted rehabilitation interventions to improve functional recovery in this vulnerable patient population.

## Introduction

The prevalence of osteoporosis is increasing in parallel with the aging of the global population. Furthermore, fractures associated with osteoporosis are also increasing, with 175,700 hip fractures reported in Japan (37,600 in men and 138,100 in women) [1]. The incidence rate in Japan is comparable to that in southern European countries and East Asian countries [2,3]. According to incidence rates by sex and age group, as well as future population estimates from 2012, the annual number of new cases is estimated to reach 290,000 in 2030 and 320,000 in 2040.

The association between osteoporotic fractures and sarcopenia has been clarified in previous studies, and a correlation between the prevalence of sarcopenia and osteoporotic fractures has been discovered [4]. The concept of sarcopenia was originally proposed in 1989, and the European Working Group on Sarcopenia in Older People (EWGSOP) published a consensus treatise that defined the definition and diagnostic criteria for sarcopenia in 2010 [5]. However, because Asians have a different average body size and composition from those typical of Caucasians, the Asian Working Group for Sarcopenia (AWGS) has established specific diagnostic criteria for Asians [6].

Past studies have reported on the relationship between sarcopenia and rehabilitation, but the total number of subjects was limited, and the follow-up period was often short. A few studies have been conducted to determine whether sarcopenia is a predictor of post-surgery rehabilitation in the field of orthopedics. Several studies have investigated whether there is a difference in activities of daily living (ADL) between patients with and without sarcopenia after hip fracture surgery [7-9]. It has been shown that patients with sarcopenia had significantly worse physical and cognitive outcomes at both admission and discharge than patients without sarcopenia [10]. However, evidence remains limited regarding whether sarcopenia can be considered a predictor of comprehensive functional recovery in orthopedic patients following surgery.

Therefore, the purpose of this study was to clarify the changes in a comprehensive set of outcomes, including balance, physical pain, ADL, walking ability, and body composition, and to examine the differences in these outcomes between patients with and without sarcopenia after hip fracture surgery. We hypothesized that patients with sarcopenia would demonstrate significantly poorer functional outcomes at discharge, including lower scores on the Berg Balance Scale (BBS) and a slower walking speed, compared to non-sarcopenic patients, even after undergoing rehabilitation.

This manuscript was previously uploaded as a preprint and is available at Research Square (https://www.researchsquare.com/article/rs-3820170/v1) [11].

## Materials and methods

Participants

This study was conducted at Shiobara Spring Hospital in Nasushiobara, Tochigi, Japan, and included patients with postoperative hip fractures admitted between October 1, 2016, and March 31, 2021. Hip fractures included femoral neck fractures and femoral trochanteric fractures. Inclusion criteria for participants were as follows: age over 65 years and the ability to walk before the fracture. Patients with dementia, pathological fractures, no measure of fitness, numerous fractures after multiple traumas, patients undergoing dialysis, and those with paralysis or significant declines in ADL after stroke were excluded from the study. Other exclusion criteria included those who were only evaluated at the time of admission or discharge and those who were not evaluated for skeletal muscle mass index (SMI). Patients who were admitted to an acute care hospital for >60 days after hip fracture surgery were also excluded because public medical insurance did not cover acute care or rehabilitation beyond this duration. The study protocol was approved by the Institutional Review Board of Shiobara Spring Hospital (approval no. 2023-1), and all patients gave detailed written informed consent. Our convalescent rehabilitation ward was able to provide inpatient rehabilitation for 40 minutes three times per day (total of 120 minutes) for approximately three months after the patient was postoperatively transferred from an acute care hospital. The physical therapists changed the rehabilitation program gradually, mainly according to the patient’s postoperative condition. The general structure and components of the program typically included a focus on gait training, balance, and strengthening exercises. We have confirmed that robotics was not used in the program.

Data collection

To ensure consistency and accuracy, all assessments were conducted by trained and experienced physical therapists at two specific time points: within 48 hours of patient admission and within 48 hours before discharge. At the time of admission, grip strength was measured on both sides, with the patient seated on the bed, elbows extended, and wrists in a neutral position. A total of three measurements were taken for each hand, and the best value was adopted. Walking speed was measured using a 10-meter walk test. The time taken for the patient to walk 10 meters at their fastest possible pace was recorded, and the walking speed was calculated in meters per second (m/s). For patients unable to perform the test, their gait speed was recorded as 0 m/s. The Barthel Index, a comprehensive index for activities of daily living (ADL), was used to evaluate a total of 10 items: fecal incontinence, urinary incontinence, and the degree of assistance needed for grooming, toilet use, feeding, transfers, walking, dressing, climbing stairs, and bathing. Each item is weighted differently, with scores ranging from 0 to 5, 0 to 10, or 0 to 15 points depending on the level of independence. The BBS was used to provide a comprehensive assessment of balance function. The BBS consists of 14 items, each scored on a five-point scale, with a maximum total score of 56 points. A higher BBS score indicates better balance ability. Patients evaluated their average lower extremity pain using an 11-point Numerical Rating Scale (NRS) (0 = no pain; 10 = worst pain imaginable) at both rest and during ambulation. Body mass index (BMI) was calculated as weight/height² (kg/m²). Calf circumference was measured at the thickest point on both legs using a non-stretchable tape measure. The mean of the right and left measurements was then calculated. Walking speed, Barthel Index, BBS, NRS, and calf circumference were evaluated at both admission and discharge to track changes over the course of the rehabilitation period.

Assessment of sarcopenia

Participants were classified as having sarcopenia if they had low SMI, low grip strength, and low walking speed, as described by AWGS 2019 [12]. Low grip strength was <28 kg for men and <18 kg for women. For both men and women, <1.0 m/s was considered a low walking speed. A multi-frequency validated bioelectrical impedance analysis (BIA) instrument, the Inbody S10 (Biospace, Seoul, Korea), was used to examine the patient in a supine position. SMI, phase angle (PhA), extracellular water (ECW), and total body water (TBW) were obtained using BIA. The ratio of ECW to TBW (ECW/TBW) was then calculated to compare the distribution of body water. The cutoff points of the SMI measured using BIA were <7.0 kg/m^2^ for men and <5.7 kg/m^2^ for women. This study involved two groups: a sarcopenia group and a non-sarcopenia group, and the following subjects were compared.

Statistical analysis

The Shapiro-Wilk test was performed as a test of normality to determine whether the samples belonged to a normal distribution. From there, the t-test was performed if the distribution was normal and the Mann-Whitney U-test if it was not normal, and the significant probability was calculated. The length of hospital stay and NRS on admission were compared using an unpaired t-test. Other items were compared using a Mann-Whitney U-test. The chi-square test was used for the analysis of categorical data. A multivariate logistic regression model was used to calculate adjusted odds ratios (ORs) with 95% confidence intervals (CIs) for factors related to sarcopenia. Continuous data are presented as mean ± standard deviation. A P value <0.05 was considered statistically significant. IBM SPSS Statistics for Windows, Version 23.0 (IBM Corp., Armonk, NY) software was used for all statistical analyses.

Ethical approval

This retrospective case-control study was performed at a single rehabilitation hospital. The study was conducted in accordance with the principles of the Declaration of Helsinki and was approved by the Ethics Review Board of Shiobara Spring Hospital (2023-1). For patients admitted after hip fractures, the evaluation on admission and discharge was compared between the groups with and without sarcopenia.

## Results

**Figure 1:**
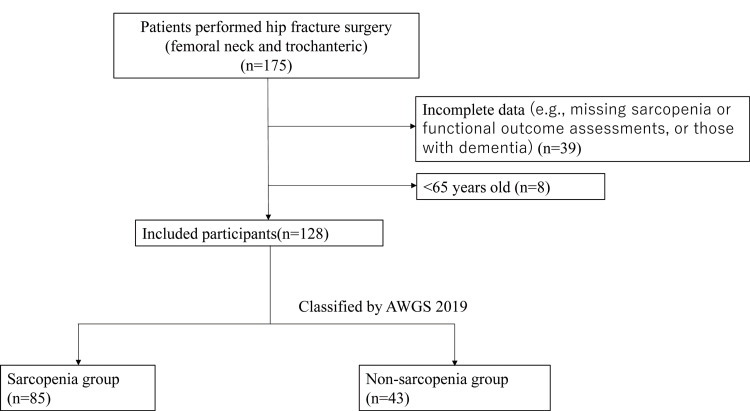
Flow chart presenting the study subjects Excluding patients who met the exclusion criteria, the total number of subjects in this study was 128. In addition, 85 were sarcopenic and 43 were non-sarcopenic.

The total number of postoperative proximal femoral fracture patients was 175. We excluded 39 patients with incomplete data and eight patients who were less than 65 years old. The total number of patients who met the criteria in this study was 128. Of the hip fractures, 68 were neck of femur fractures and 60 were femoral transverse fractures. There was no significant difference in fracture type between the sarcopenia and non-sarcopenia groups. The patients were treated with one total hip arthroplasty, 55 bipolar hemiarthroplasties, and 72 internal fixations. Sarcopenia status during admission for postoperative hip fracture was determined in 128 participants; 85 (66.4%) had sarcopenia, and 43 (33.6%) did not have sarcopenia (Figure 1).

Table 1 shows the differences between the sarcopenia group and the non-sarcopenia group at admission. There was no significant difference in age between the sarcopenia group and the non-sarcopenia group (87.0 ± 6.8 vs. 81.0 ± 9.6 years, respectively) (Table 1). Similarly, the sarcopenia group was significantly lower in height, weight, BMI, hand grip, SMI, and PhA than the non-sarcopenia group. The length of hospital stay was significantly longer in the sarcopenia group compared to the non-sarcopenia group. In the ECW/TBW ratio, the sarcopenia group was significantly higher than the non-sarcopenia group.

**Table 1:** Characteristics of patients with and without sarcopenia in a convalescent rehabilitation ward P-values were calculated using t-test or Mann–Whitney U-test for continuous variables and chi-square test for categorical variables. BMI, body mass index; SMI, skeletal muscle mass index; ECW/TBW, extracellular water/total body water *P < 0.05

	Total (n = 128)	With sarcopenia (n = 85)	Without sarcopenia (n = 43)	p-value
Age (years)	85.0±8.3	87.0±6.8	81.0±9.6	0.081
Sex, n (%)	-	-	-	-
Female	95 (74)	65 (76)	30 (70)	0.413
Male	33 (26)	20 (24)	13 (30)	-
Height (cm)	151.3±9.6	149.4±8.2	155.1±10.4	0.007*
Weight (kg)	47.1±9.7	44.1±7.5	52.8±11.0	0.002*
BMI (kg/m^2^)	20.4±3.2	19.8±2.9	21.8±3.4	0.015*
Handgrip strength (kg)	14.7±6.6	13.1±4.7	17.9±8.4	0.007*
Length of hospital stay (days)	75.6±25.8	79.7±24.3	67.7±27.1	0.012*
Number of days until transfer after surgery (days)	28.4±13.1	27.5±13.2	29.9±13.0	0.271
SMI (kg/m^2^)	5.5±1.1	4.9±0.8	6.6±1.1	<0.001*
ECW/TBW ratio	0.413±0.017	0.416±0.009	0.407±0.027	0.044*
Phase angle	3.52±0.72	3.33±0.54	3.93±0.87	0.001*

**Figure 2:**
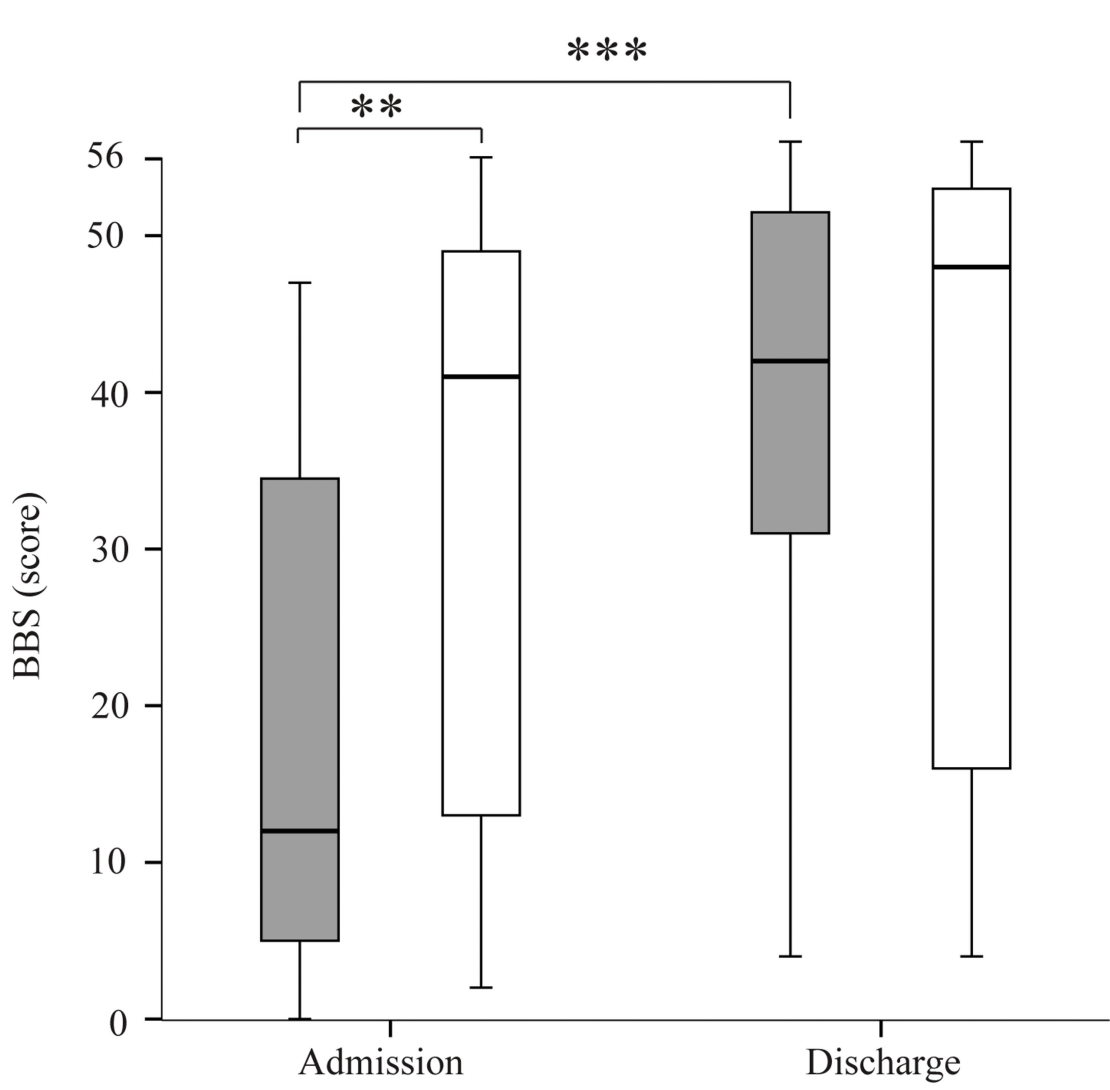
Comparison of the Berg Balance Scale (BBS) scores between the sarcopenia and non-sarcopenia groups on admission and at discharge Gray boxes represent the sarcopenia group; white boxes represent the non-sarcopenia group. *p < 0.05, **p < 0.005, ***p < 0.001

The BBS in the sarcopenia group was higher on discharge than on admission, but in the non-sarcopenia group, there were no significant differences between discharge and admission (Figure 2). The BBS on admission and on discharge was significantly higher in the non-sarcopenia group than in the sarcopenia group.

**Figure 3:**
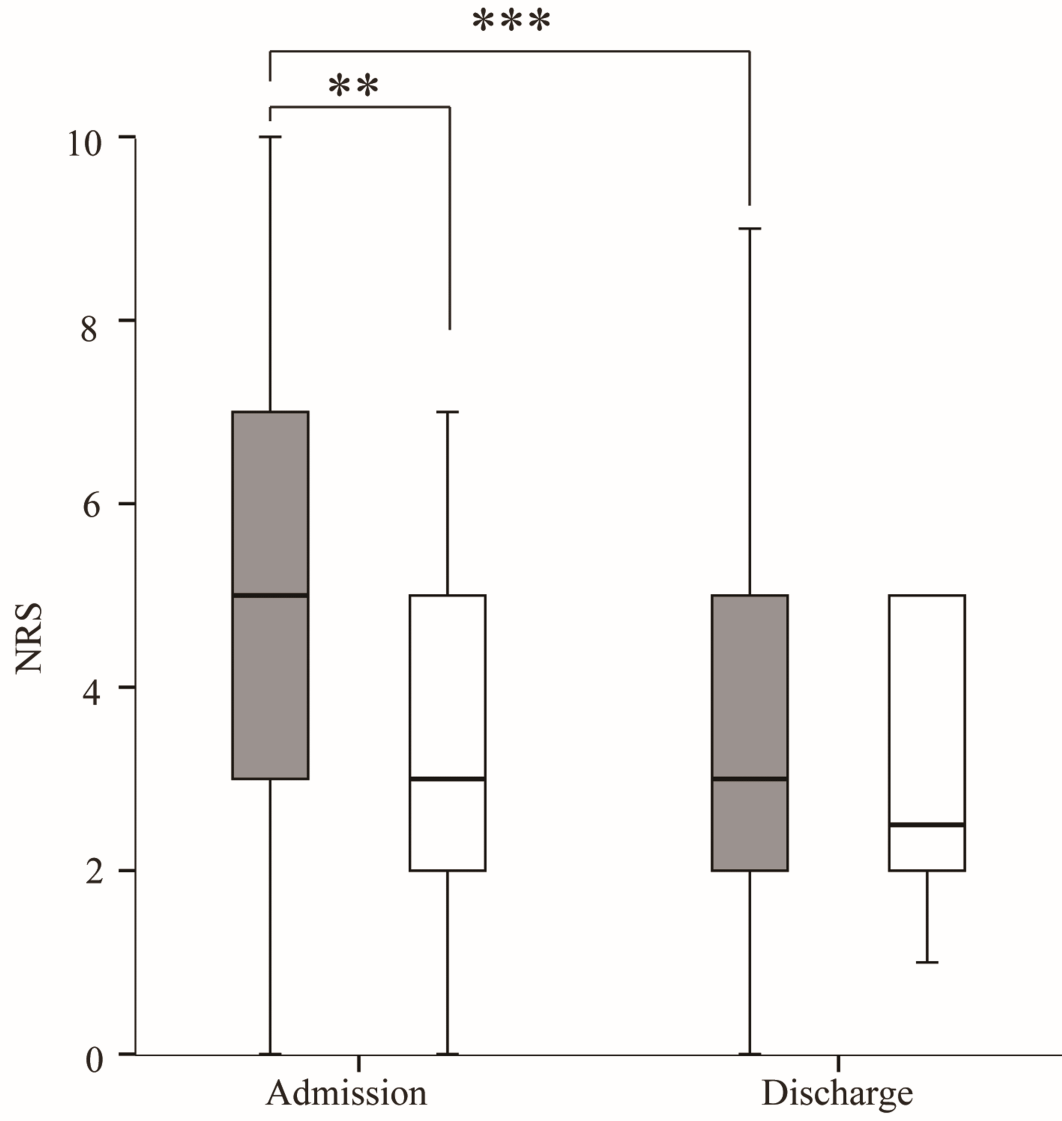
Comparison of the numerical rating scale (NRS) scores between the sarcopenia and non-sarcopenia groups on admission and at discharge Gray boxes represent the sarcopenia group; white boxes represent the non-sarcopenia group. *p < 0.05, **p < 0.005, ***p < 0.001

The NRS on discharge was significantly lower in the sarcopenia group than on admission (Figure 3). In the non-sarcopenia group, the NRS showed no significant difference between discharge and admission. The NRS on admission was significantly higher in the non-sarcopenia group than in the sarcopenia group, but the NRS on discharge showed no significant differences.

**Figure 4:**
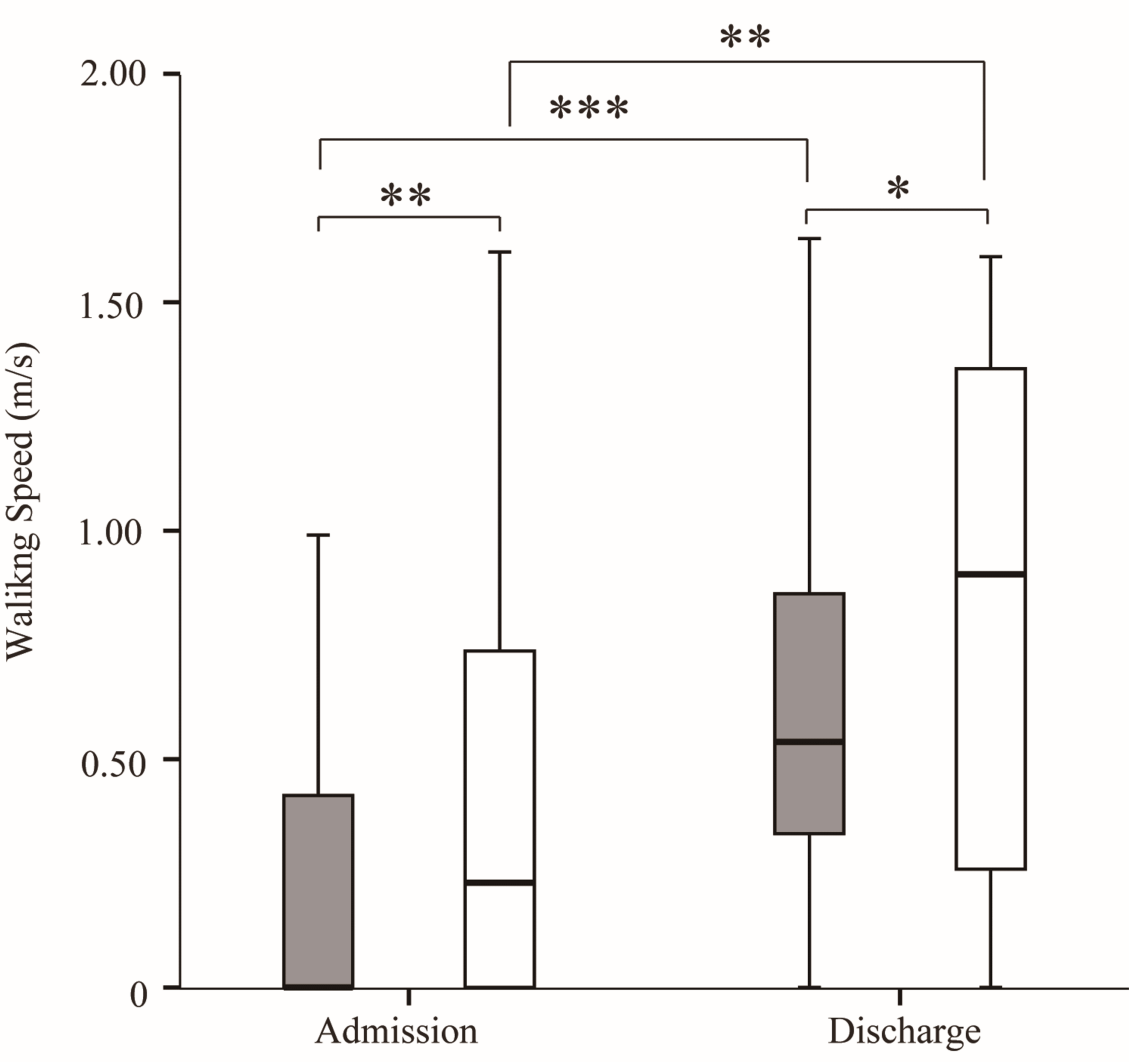
Comparison of walking speed between the sarcopenia and non-sarcopenia groups on admission and at discharge Gray boxes represent the sarcopenia group; white boxes represent the non-sarcopenia group. *p < 0.05, **p < 0.005, ***p < 0.001

Walking speeds on discharge were faster than on admission in both groups (Figure 4). Both on admission and on discharge, the walking speed in the non-sarcopenia group was significantly faster than in the sarcopenia group.

**Figure 5:**
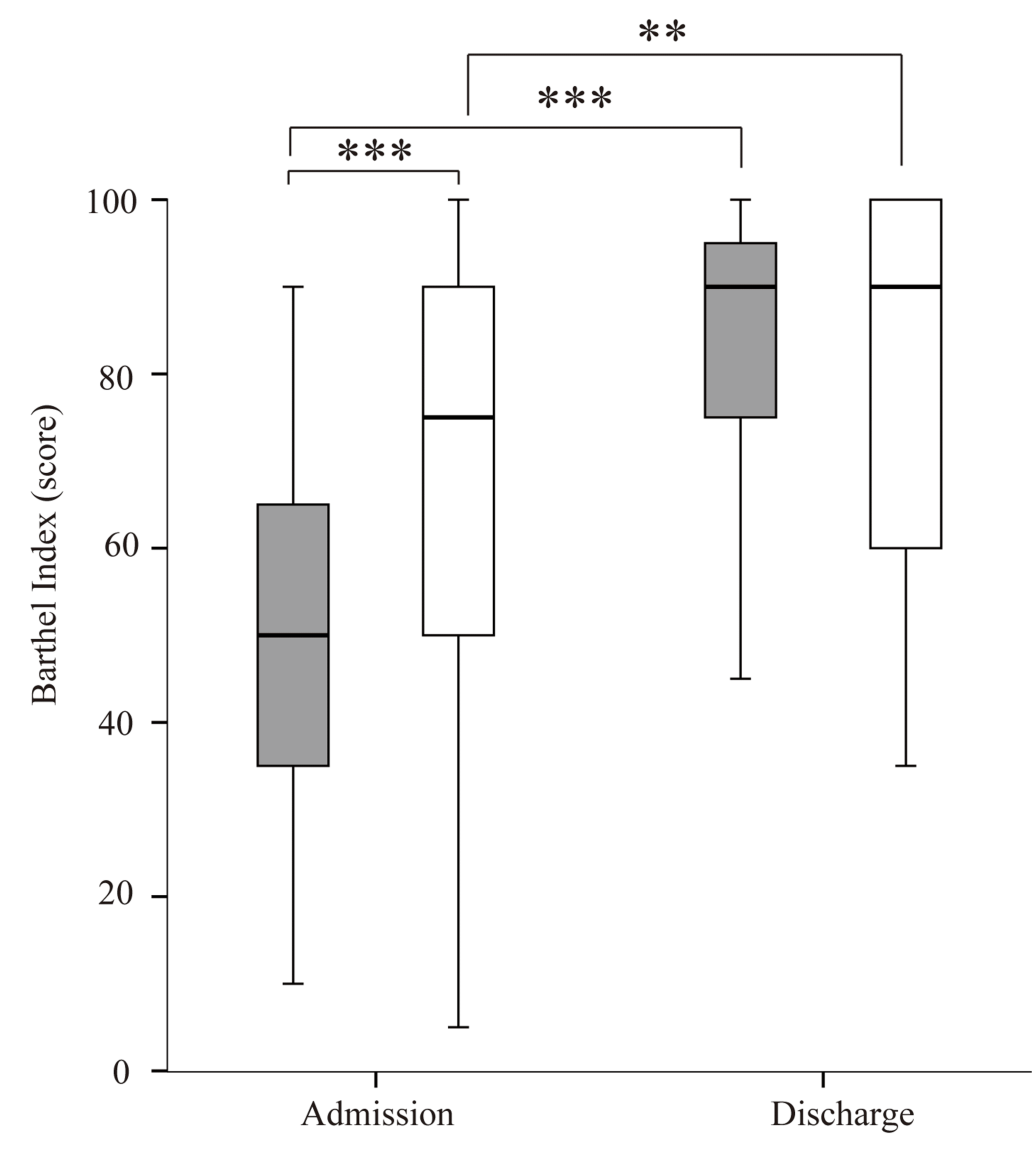
Comparison of the Barthel Index between the sarcopenia and non-sarcopenia groups on admission and at discharge Gray boxes represent the sarcopenia group; white boxes represent the non-sarcopenia group. *p < 0.05, **p < 0.005, ***p < 0.001

The Barthel Index was higher on discharge than on admission in both groups (Figure 5). The Barthel Index on admission was significantly higher in the non-sarcopenia group than in the sarcopenia group. The Barthel Index on discharge was significantly higher in the non-sarcopenia group than in the sarcopenia group.

**Figure 6:**
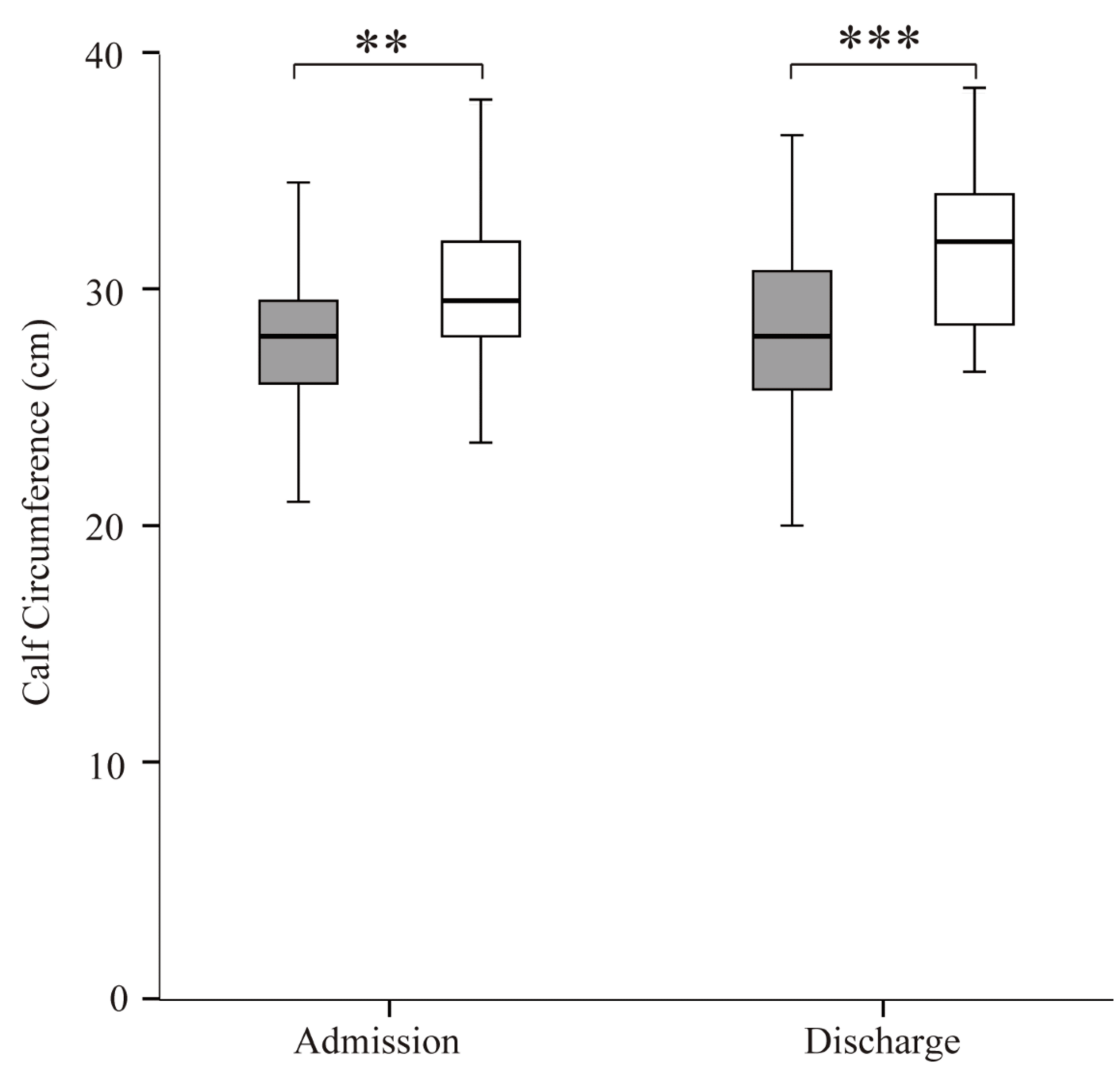
Comparison of calf circumference between the sarcopenia and non-sarcopenia groups on admission and discharge Gray boxes represent the sarcopenia group; white boxes represent the non-sarcopenia group. *p < 0.05, **p < 0.005, ***p < 0.001

The calf circumference was significantly higher in the non-sarcopenia group than in the sarcopenia group both on admission and on discharge (Figure 6). In both groups, the calf circumference showed no significant difference between discharge and admission.

Multivariate logistic regression analysis was performed to identify factors associated with sarcopenia at admission and discharge, while adjusting for age and gender. On admission, the presence or absence of sarcopenia did not show a statistically significant association with dependent variables such as BBS, NRS, and Barthel Index. However, on discharge, sarcopenia was significantly associated with both calf circumference (OR 1.429, 95% CI 1.145-1.784, P = 0.002) and walking speed (OR 28.635, 95% CI 4.780-171.552, P < 0.001).

## Discussion

In this study, we investigated the prevalence of sarcopenia in patients with hip fractures after surgery and confirmed its relationship with balance and lower extremity pain. Our findings revealed that, among the 128 enrolled patients, sarcopenia was present in 85 (66.4%). Moreover, the Barthel Index and BBS in the sarcopenia group improved more on discharge than on admission.

The prevalence of sarcopenia in hip fracture patients ranged from approximately 17% to 70% in previous studies [8,10,13,14]. There was wide variation in the prevalence of sarcopenia, ranging from 12% to 95% in men and 18% to 68% in women [8,13,14]. Other reports have focused on the prevalence of sarcopenia in the acute phase, whereas the present study focused on the subacute phase after surgery at a different hospital, which may have altered the percentages. In our study, the percentage of female patients was higher (68.4%) than that of men (60.6%), with a 66.4% overall prevalence of sarcopenia. The prevalence of sarcopenia was comparable in our study compared with previous reports.

The BBS, a comprehensive balance assessment tool, was administered by trained and experienced physical therapists to measure balance and to serve as an indicator of the need for a walker [15,16]. Kobayashi et al. [15] reported moderate areas under the receiver operating characteristic curve (AUCs) of 0.824 and 0.865 for predicting the ability to walk without aids and with a walker, respectively, with cutoff values of 51.5 and 45.5 points. Tamura et al. showed that the cutoff values of the BBS score on admission to predict independent and supervised walking on discharge were 28 (AUC = 0.76) and 21 (AUC = 0.84) points, respectively [16]. In this study, the sarcopenia group had a lower BBS score on admission than the non-sarcopenia group. Both the sarcopenia and non-sarcopenia groups had better BBS scores on discharge after rehabilitation (38.4 ± 14.6 vs. 37.0 ± 19.3) than at admission (18.9 ± 15.8 vs. 33.1 ± 18.2).

The current study results showed that the sarcopenia group improved significantly from an NRS of 5.3 ± 2.2 on admission to 3.5 ± 2.5 on discharge. The non-sarcopenia group improved from an NRS of 3.5±1.8 on admission to 3.1±1.5 on discharge. Arinzon et al. showed that the average VAS score in elderly patients with hip fractures on admission was 7.38 ± 1.20 and 3.67 ± 1.18 after rehabilitation treatment [17]. Although the NRS in the sarcopenia group was significantly higher at admission than in the non-sarcopenia group, both groups improved after rehabilitation, thus indicating that rehabilitation was effective.

In a previous report, compared with patients without sarcopenia, patients with sarcopenia after hip fracture had a lower Barthel Index score on admission to the rehabilitation unit (31.3 vs. 28.0, respectively, p = 0.28), at discharge (69.2 vs. 58.9, respectively, p < 0.001), and after three-month follow-up (90.9 vs. 80.5, respectively, p = 0.02) [18]. The Barthel Index, a comprehensive ADL index, was administered by experienced physical therapists to assess functional independence. This study showed that patients with sarcopenia had a lower Barthel Index score on admission (50.0 vs. 66.3, respectively; p < 0.001) and at discharge (66.3 vs. 80.0, respectively; p = 0.754) than patients without sarcopenia. The higher Barthel Index scores in this study compared with previous studies may be caused by the longer hospitalization period in Japan compared with other countries.

Calf circumference in the non-sarcopenia group was higher than in the sarcopenia group both on admission and on discharge. Calf circumference has been shown to predict nutritional risk [19], disability [20], mortality [21], bone mineral density [22], and sarcopenia [23]. Borges et al. showed that calf circumference had a relatively high predictive capacity for sarcopenia and could be a valuable tool for predicting the risk of sarcopenia compared with currently used screening tools [23]. In this study, calf circumference in both the sarcopenia and non-sarcopenia groups showed no significant difference between admission and discharge because recovery may require more time than rehabilitation.

Previous studies have shown that the PhA in patients with hip fractures was significantly lower than that in healthy older adults [24]. In the present study, patients who developed sarcopenia after hip fracture surgery showed a lower PhA and higher ECW/TBW than patients without sarcopenia. ECW/TBW is the ratio of extracellular fluid to body water content and is elevated in edema-causing diseases such as dialysis and heart failure [25]. PhA reflects the physiological level of cells and has been reported to be a prognostic predictor, especially in oncology, gastroenterology, and cardiovascular medicine [26-28]. PhA has been reported to be a predictor of prognosis in cancer patients with sarcopenia [27,28], but it is unclear whether PhA is a prognostic factor for hip fracture patients with sarcopenia. In this study, PhA was significantly lower in the sarcopenia group, and we speculate that it may be a predictor of prognosis in hip fracture patients.

The multivariate logistic regression analysis reveals that sarcopenia is a significant independent predictor of poorer physical outcomes at discharge, even after adjusting for age and gender. While no significant associations were observed with baseline functional scores (BBS, NRS, Barthel Index) at admission, a clear link emerged by the time of discharge, suggesting that the detrimental effects of sarcopenia are exacerbated during hospitalization. The particularly high odds ratio for walking speed (OR 28.635) is a compelling finding, indicating that sarcopenic patients are over 28 times more likely to have a slower walking speed at discharge compared to their non-sarcopenic counterparts. Given that walking speed is a robust indicator of frailty and future mortality [29], this result underscores the profound clinical importance of sarcopenia. Conversely, the smaller but still significant odds ratio for calf circumference (OR 1.429) suggests a less direct association with functional decline. Our findings align with previous research highlighting the link between sarcopenia and negative outcomes such as longer hospital stays and increased readmission rates [30]. Future research should investigate the effectiveness of such interventions, including nutritional support and physical therapy, to improve discharge outcomes for sarcopenic patients.

The present study has several limitations that should be considered when interpreting the results. First, as a retrospective, single-center observational study, it is challenging to establish a definitive causal relationship between sarcopenia and rehabilitation outcomes. The study design also prevents us from collecting crucial data on patients' pre-fracture condition and independence, cognitive function, and the severity or type of hip fracture, all of which are known to influence rehabilitation outcomes. We also lacked detailed information on early postoperative complications and specific treatment details, such as analgesic use, which could have been important confounding factors. While we provided a partial breakdown of fracture types and compared treatment modalities, we acknowledge that a prospective study is necessary to collect these crucial data and draw more definitive conclusions.

Second, we recognize that the assessment and diagnosis of sarcopenia in the acute, early postoperative period is challenging. Postoperative pain and the acute inflammatory response can significantly affect physical performance measures like walking speed. It is therefore conceivable that some patients may have been temporarily classified as sarcopenic due to pain-related gait impairment, which could potentially confound the between-group comparisons. This potential for misclassification is a significant limitation.

Third, the observational period was limited to approximately 2-3 months, which may not be sufficient to fully assess the long-term effects of rehabilitation. Although our findings demonstrate the short-term benefits of rehabilitation, future studies should evaluate 1- or 2-year outcomes to assess the lasting impact.

Fourth, our exclusion criteria, including patients with dementia, pathological fractures, severe comorbidities, and those with prolonged acute care hospitalizations, may have introduced selection bias. While these criteria were necessary to ensure consistent functional assessment and reflect constraints of the Japanese national health insurance system, they may limit the generalizability of our findings to more complex or frail patients.

Finally, while our results showed significant improvements in the sarcopenia group with rehabilitation, we acknowledge that their absolute functional scores, such as the BBS, remained lower than the non-sarcopenia group at discharge. This suggests that the recovery potential of the two groups may not be equal. Moreover, a ceiling effect might have occurred with the BBS, where some patients in the sarcopenia group reached the maximum score of 56. This could have limited our ability to statistically detect further improvements and may have obscured the true recovery potential of these patients.

## Conclusions

Our study provides compelling evidence that sarcopenia, diagnosed at admission, serves as a significant independent predictor of poorer functional outcomes at discharge for patients undergoing rehabilitation after hip fracture surgery. A multivariate logistic regression analysis confirmed a powerful association between sarcopenia and a substantially slower walking speed at discharge. This is a crucial finding, as walking speed is a well-established and robust indicator of overall health and future mortality.

While sarcopenic and non-sarcopenic patients both showed improvements in balance and pain during rehabilitation, the sarcopenic group began their recovery from a significantly lower functional baseline. This suggests that sarcopenia is not merely a condition of low muscle mass but a key determinant of functional decline in a clinical setting.

In conclusion, our results highlight the clinical importance of early identification of sarcopenia and the need for targeted rehabilitation strategies to improve discharge outcomes for this vulnerable patient population.
